# A Review of Dual-Emission Carbon Dots and Their Applications

**DOI:** 10.3390/molecules28248134

**Published:** 2023-12-17

**Authors:** Jing Ma, Lingbo Sun, Feng Gao, Shiyu Zhang, Yuhan Zhang, Yixuan Wang, Yuecheng Zhang, Hongyan Ma

**Affiliations:** 1 Key Laboratory of Analytical Technology and Detection of Yan’an, College of Chemistry and Chemical Engineering, Yan’an University, Yan’an 716000, China; majing@yau.edu.cn (J.M.); 18691095199@163.com (S.Z.); yxwang_yau@163.com (Y.W.); mahy6614@yau.edu.cn (H.M.); 2 Medical College of Yan’an University, Yan’an University, Yan’an 716000, China; lingbosun@yau.edu.cn (L.S.); z13201471702@outlook.com (Y.Z.); 3 Xi’an Zhongkai Environmental Testing Co., Ltd., Xi’an 710000, China; 18092780503@163.com

**Keywords:** dual-emission carbon dots, photoluminescence mechanism, ratiometric sensing, bioimaging

## Abstract

Carbon dots (CDs), as a rising star among fluorescent nanomaterials with excellent optical properties and fascinating dual-emission characteristics, have attracted increasing attention in sensing, bio-imaging, drug delivery, and so on. The synthesis of dual-emission CDs (DE-CDs) and the establishment of ratiometric fluorescence sensors can effectively diminish background interference and provide more accurate results than single-emission CDs. Although DE-CDs have generated increased attention in many fields, the review articles about DE-CDs are still insufficient. Therefore, we summarized the latest results and prepared this review. This review first provides an overview of the primary synthesis route and commonly used precursors in DE-CDs synthesis. Then, the photoluminescence mechanism behind the dual-emission phenomenon was discussed. Thirdly, the application of DE-CDs in metal cation detection, food safety analysis, biosensing, cell imaging, and optoelectronic devices has been extensively discussed. Finally, the main challenges and prospects for further development are presented. This review presents the latest research progress of DE-CDs synthesis and its application in ratiometric sensing; hopefully, it can help and encourage researchers to overcome existing challenges and broaden the area of DE-CDs research.

## 1. Introduction

Carbon dots (CDs) were first discovered in 2004 by Xu et al. when separating and purifying single-walled carbon nanotubes through electrophoresis [1]. However, the wide interest in CDs was stimulated by Sun et al. in 2006 when this new carbon material was formally defined as “carbon dots” [2]. Actually, CDs are generally a group of quasi-spherical carbon nanoparticles, with diameters ranging from 1 to 10 nm, consisting of sp^2^/sp^3^ carbons, are oxygen/nitrogen based, and feature other modified chemical groups [3,4]. As a new member of carbon materials, CDs have become a research hotspot during the past decade because of their numerous merits, including excellent biological compatibility, fascinating optical properties, good water solubility, and low cellular toxicity [5,6]. Additionally, CDs can be simply and economically synthesized through the “Top-down” and “Bottom-up” approaches by using various C-containing materials as precursors [7,8]. Based on the unique properties stated above, CDs have received considerable attention and have been widely applied in sensing [9,10,11], bio-imaging [12,13], drug delivery [14,15], optoelectronic devices [16,17], and so on.

As we know, the surfaces of CDs contain a rich number of different functional groups that can provide abundant binding sites for the specific recognition of targets [18]. In a typical sensing process, the interaction between the target and CDs would cause a change in the optical signal of CDs, such as the fluorescence intensity or a shift in emission wavelength, which may directly provide information for the quantification of the target [9,19]. If the target molecule cannot influence the fluorescence signal of CDs, then CDs cannot be applied as a sensor for this target. However, most of the current sensing and quantification processes rely on the enhancing or quenching signal from a single wavelength, which makes the quantification fraught with difficulties since it can be affected by many interferences, including the instrumental parameters, the local concentration of the target, the microenvironment, photobleaching, and so on [20,21]. The ratiometric assay is one of the best ways to solve this problem [22]. In a ratiometric approach, the quantification process relies on target-induced changes from at least two emission bands, which would provide an effective internal reference to increase the sensitivity, stability, and accuracy [21,23].

Currently, there are three main routes to construct CD-based ratiometric fluorescence probes: physical mixing, nanohybrid, and dual-emission CDs [24,25]. The physical mixing strategy is a quick and facile way to establish a CD-based ratiometric probe by simply mixing as-synthesized CDs with other optical materials, including quantum dots (QDs), metal nanoclusters, dyes, and other CDs [26,27,28,29]. Castro and co-workers established a ratiometric sensor for ibandronic acid detection by mixing blue-emitting CDs with orange-emitting AgInS_2_ QDs [27]. Since ibandronic acid can only quench the orange emission from AgInS_2_ QDs, the blue emission from CDs and the orange emissions from AgInS_2_ QDs can be used as control and responsive signals for the ratiometric detection of ibandronic acid. In the nanohybrid strategy, CDs are combined with other optical materials covalently or non-covalently to form PL nano-assemblies with dual emission [30,31,32,33,34]. Shen’s group prepared a ratiometric probe for tetracycline by combining blue-emitting CDs with Eu^3+^. As we know, Eu^3+^ could coordinate with carbonyl and amino groups on the surface of CDs and exhibit a quenching state of Eu^3+^. In the presence of tetracycline, a CD-Eu^3+^-tetracycline ternary complex is formed, generating an intense red emission from Eu^3+^ [35]. Though these methods can provide practical ways to establish ratiometric sensors, they still need another optical material, significantly complicating the synthesis procedure. The dual-emission CD strategy was based on the direct dual emission from CDs without further addition of luminescent materials [36,37,38]. Therefore, this strategy provides a simple and accurate approach to establishing ratiometric probes which call for the direct synthesis of dual-emission carbon dots (DE-CDs) and establishing an effective ratiometric sensor [39,40,41,42].

**Figure 1:**
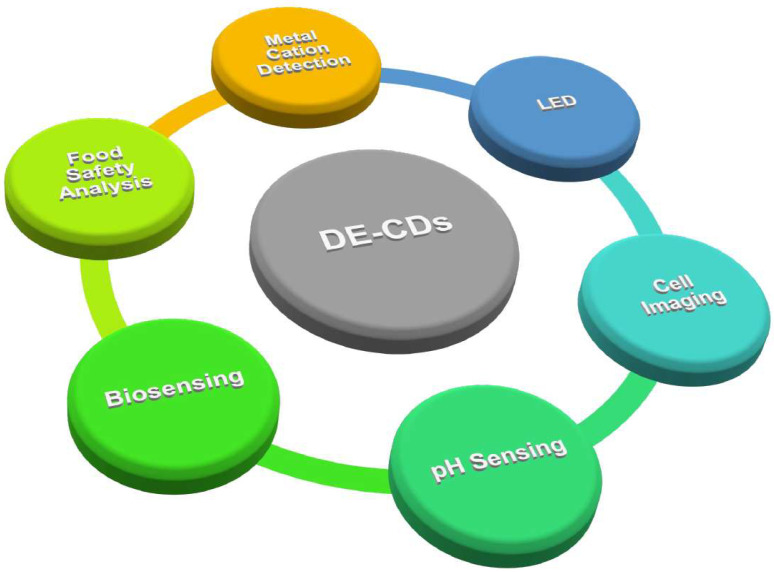
Various applications of DE-CDs.

Previous reviews have provided a detailed overview of the synthesis strategies, basic mechanisms, and different applications of CDs in sensing and bioimaging; however, a detailed overview of DE-CDs is still lacking [43,44,45,46]. Therefore, we would like to review the synthesis and application of DE-CDs in recent years. This review first provides an overview of the commonly used synthesis route and precursor for the fabrication of DE-CDs. Then, the photoluminescence (PL) mechanism behind the dual-emission phenomenon is discussed. The application of DE-CDs in metal cation detection, food safety analysis, biosensing, cell imaging, and optoelectronic devices is further summarized (Figure 1). This review is intended to help researchers understand, and to provide overall information on, DE-CDs and their application as DE-CD-based ratiometric sensors.

## 2. Preparation of DE-CDs

### 2.1. Preparation Methods for DE-CDs

CDs can be synthesized through a variety of methods, which can be classified into two categories: “top-down” and “bottom-up” [47]. The top-down synthesis method involves decomposing larger pieces of carbon structures, such as graphene, carbon nanotubes, and activated carbon, through techniques including arc discharge, electrochemical oxidation, and laser ablation [48,49]. In 2007, Zhou et al. proposed the electrochemical synthesis method to convert multi-walled carbon nanotubes to highly efficient blue luminescent carbon nanocrystals [50]. However, these approaches usually require a long preparation time, poor reaction conditions, and expensive materials and equipment [51]. Considering these harsh experimental conditions, fewer reports currently use top-down methods to generate CDs [52]. In contrast to the top-down approach, CDs can also be obtained from bottom-up processes by carbonizing small carbon-containing precursors. The carbon-containing molecules can be amino acids, synthetic polymers, and biomass. Many approaches can be used for carbonization, including hydrothermal/solvothermal carbonization, microwave/ultrasonication, combustion, and pyrolyzation. Yang et al. reported a simple and effective synthesis method via the hydrothermal treatment of glucose to synthesize fluorescent CDs in the presence of potassium dihydrogen phosphate [53]. Zhu et al. synthesized fluorescent carbon-based nanoparticles with electrochemical luminescence properties through microwave pyrolysis [54]. Because of the high efficiency and convenience of these methods, bottom-up methods, especially hydrothermal and solvothermal approaches, are widely used nowadays.

**Figure 2:**
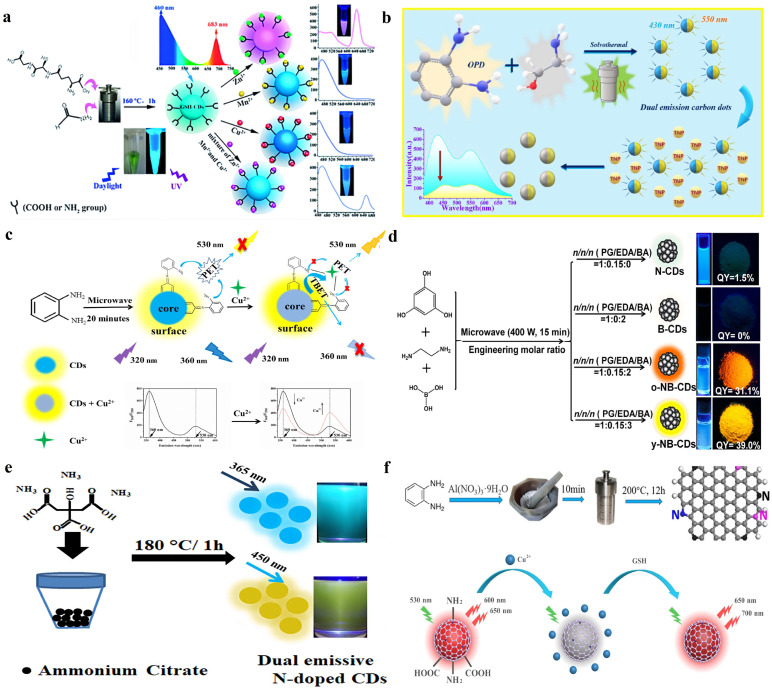
(**a**) Solvothermal synthesis of DE-CDs and their application for multiple detection of heavy metal ions. Reproduced with permission from ref. [56], copyright 2018, The WILEY-VCH Verlag GmbH & Co. KGaA. (**b**) Solvothermal synthesis of DE-CDs and their application in the ratiometric TNP sensor. Reproduced with permission from ref. [57], copyright 2022, The Elsevier. (**c**) Microwave-assisted synthesis of DE-CDs and their application in copper detection. Reproduced with permission from ref. [38], copyright 2021, The Elsevier Ltd. (**d**) Microwave-assisted synthesis and photographs of DE-CDs in solution and powder under UV light. Reproduced with permission from ref. [58], copyright 2021, The American Chemical Society. (**e**) N-doped DE-CDs were synthesized through simple annealing of ammonium citrate in the air via solvent-free carbonization. Reproduced with permission from ref. [59], copyright 2022, The Elsevier Ltd. (**f**) Preparation of red DE-CDs by a one-step pyrolysis method. Reproduced with permission from ref. [60], copyright 2021, The Elsevier B.V.

The hydrothermal/solvothermal method is one of the most popular bottom-up methods. During the hydrothermal/solvothermal process, driven by high temperature and high vapor pressures, small organic molecules start to be continuously dehydrated in water or other solvents to form polymer clusters. Further, they carbonize to create DE-CDs [55]. Due to the large variety of precursors and simple/environmentally friendly synthesis process, the hydrothermal/solvothermal method is widely used in DE-CDs synthesis. For instance, Yu et al. synthesized cyan fluorescent DE-CDs through the solvothermal treatment of glutathione (GSH) and formamide at 160 °C for 1 h [56]. As shown in Figure 2a, the obtained DE-CDs exhibit dual emission bands at 460 and 683 nm when excited with ultraviolet light (λ = 365 nm), which can be further applied for the multiple detection of heavy metal ions. Wang et al. reported a simple and effective method for the preparation of DE-CDs via the solvothermal reaction by using o-phenylenediamine (*o*-PD) and ethanolamine as raw materials [57]. Under the excitation of ultraviolet light, as-synthesized DE-CDs showed two distinguishable emission peaks at 430 and 550 nm, and they could be used as a proportional fluorescence sensor to detect 2, 4, 6-trinitrophenol (TNP) sensitively and selectively (Figure 2b).

The microwave-assisted method is a facile and fascinating strategy to generate DE-CDs since DE-CDs can be produced within a few minutes [61,62]. This is because microwaves are electromagnetic waves with frequencies ranging from 300 MHz to 300 GHz, which helps efficiently break the chemical bonds in raw materials, providing an ultra-fast procedure for DE-CD synthesis [45,62]. Huang et al. dissolved *o*-PD in 20 mL distilled water to form a transparent solution and then heated it with a household microwave oven at 700 W for 20 min [38]. After filtration and purification, Figure 2c shows that DE-CDs with dual emission at 360 and 530 nm were obtained, which can be further applied in copper detection. By using phloroglucinol dihydrate, boric acid, and ethylenediamine as raw materials, Wang’s group synthesized the novel DE-CDs with the microwave-assisted method by heating raw materials in a 400 W microwave oven (M1-235C) for 15 min [58]. Different DE-CDs with various optical properties were synthesized by changing the ratio of precursors. As shown in Figure 2d, one exhibited dual emission peaks at 484 and 565 nm with a high solid quantum yield (QY) of 39.0%, while another DE-CDs showed bright orange fluorescence (dual emission at 484 and 585 nm), with a solid-state QY of 31.1%.

Except for the traditional hydrothermal/solvothermal and microwave-assisted methods, the solvent-free carbonization method has also been used to prepare DE-CDs. Khan synthesized N-doped DE-CDs by simply annealing ammonium citrate in the air via solvent-free carbonization (Figure 2e) [59]. This method has advantages of a simple preparation, high QY, solvent-free, low cost, and the ability to generate a large number of DE-CDs. Yang et al. prepared red DE-CDs by a one-step pyrolysis method using *o*-PD as the precursor and Al(NO_3_)_3_·9H_2_O as an additive [60]. As shown in Figure 2f, the prepared DE-CDs exhibit interesting dual emissions at 600 and 650 nm, and they can be further used to determine Cu^2+^ and glutathione.

### 2.2. Commonly Used Precursors for DE-CDs Preparation

Precursors play critical roles in CD synthesis since they can directly affect the optical properties of CDs. Although thousands of precursors have been reported, the raw materials used for DE-CDs are much less than that of CDs. In this section, we would like to overview the precursors used for DE-CDs synthesis in recent years, mainly classified as aromatic small molecules, amino acids, acids, and biomass (Table 1).

**Figure 3:**
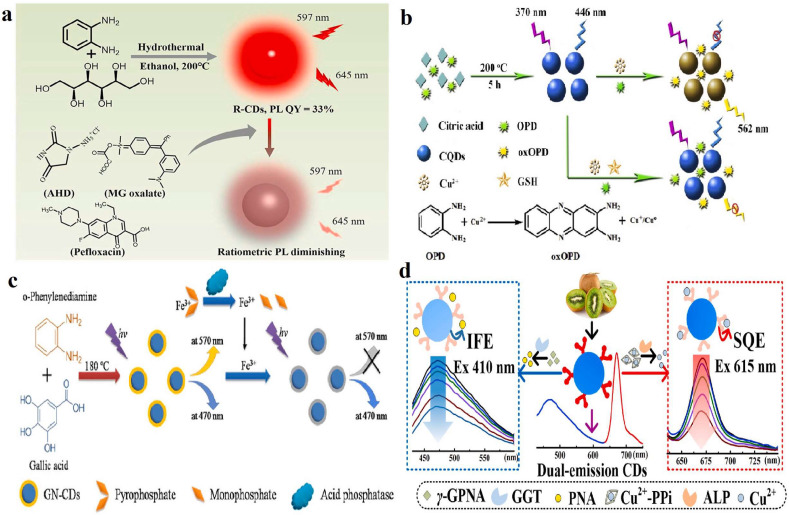
(**a**). Schematic representation of the synthesized DE-CDs and its application for the detection of veterinary drugs. Reproduced with permission from ref. [63], copyright 2021, The Elsevier B V. (**b**) Schematic of DE-CDs synthesis and principle of detection of Cu^2+^ and GSH. Reproduced with permission from ref. [64], copyright 2019, The Elsevier B.V. (**c**) Synthesis of DE-CDs and ratio assay of Fe^3+^ and acid phosphatase. Reproduced with permission from ref. [74], copyright 2020, The Elsevier B.V. (**d**) Schematic application of DE-CDs synthesis and detection of GGT and ALP activity. Reproduced with permission from ref. [83], copyright 2021, The Elsevier B.V.

Aromatic small molecules have been repeatedly reported as precursors for DE-CDs, and the most commonly used isomer is *o*-PD. For example, Li et al. prepared red DE-CDs via the solvothermal method using *o*-PD and sorbitol as precursors (Figure 3a) [63]. The obtained DE-CDs exhibit non-excitation independence dual emission at 597 and 645 nm. Interestingly, the obtained DE-CDs show quick signal response towards MG oxalate, 1-amino hydantoin hydrochloride, and pefloxacin, which could be further applied to the rapid and highly sensitive detection of these veterinary drug residues. Han et al. prepared DE-CDs (at 370 and 446 nm) via the hydrothermal method using *o*-PD and citric acid as precursors, which could be used for the quantitative fluorescence detection of Cu^2+^ and GSH (Figure 3b) [64]. Wang et al. established a simple and effective approach to prepare DE-CDs via the solvothermal reaction with *o*-PD and ethanolamine [57]. The obtained DE-CDs showed two obvious fluorescence emission peaks (430 and 550 nm) when excited with UV light. Due to the sensitive and selective reaction with TNP, a ratiometric fluorescent sensor was established, which could be used for visualizing intracellular TNP in live HeLa cells.

Amino acids are a class of organic compounds containing amino and carboxylic acid which are widely used as raw materials for CD synthesis. Until now, many amino acids, including L-cystine, L-arginine, L-tryptophan, glycine, and lysine, have been used as raw materials for DE-CDs synthesis [65,66,67,68,69,70]. Zhang et al. dissolved L-cystine and *o*-PD in 20 mL ethanol and synthesized the DE-CDs via the solvothermal method [65]. The as-synthesized DE-CDs exhibited dual emission at 595 and 648 nm, with a relatively high QY (35.7%). Mei’s group synthesized intrinsic DE-CDs co-doped with N and S atoms via the solvothermal method using L-arginine, *o*-PD, and sulfuric acid as raw materials [66]. The characterization results demonstrate that the DE-CDs were successfully synthesized, and two separate emissions peaks at 468 and 628 nm can be observed. After the interaction between the as-synthesized DE-CDs and Cr^6+^, the signal at 468 nm was significantly quenched, while the intensity at 628 nm increased. Therefore, a ratiometric sensor for Cr^6+^ was established, which could be further applied for cell imaging with dual fluorescence emission.

**Table 1:** The overview of the synthetic methods and precursors for DE-CDs.

Ref.	Synthetic Method	Precursors	Reaction Conditions	Size (nm)	Peaks (nm)
[64]	hydrothermal	*o*-PD, citric acid	200 °C, 5 h	1.59	370/446
[68]	hydrothermal	glycine, 2,4-dihydroxybenzoic acid	200 °C, 24 h	-	454/515
[69]	hydrothermal	L-tryptophan, ethylenediaminetetraacetic acid	160 °C, 6 h	9.4	360/450
[71]	hydrothermal	*m*-PD, H_2_SO_4_	200 °C, 10 h	4.3	360/520
[72]	hydrothermal	*o*-PD, phosphoric acid	200 °C, 24 h	5	440/624
[73]	hydrothermal	*o*-PD, oxalic acid	180 °C, 8 h	3.29	453/560
[74]	hydrothermal	*o*-PD, gallic acid	180 °C, 4 h	3.21	470/570
[75]	hydrothermal	*o*-PD, 2-hydroxy-3 methoxybenzaldehyde	180 °C, 8 h	2.40	430/570
[76]	hydrothermal	*o*-PD, o-aminophenol, ethanol	220 °C, 24 h	-	598 /650
[77]	hydrothermal	*o*-PD, phosphoric acid	200 °C, 24 h	4	439/630
[78]	hydrothermal	glutathione, Sodium alginate, formamide	160 °C, 2 h	3.60	480/650
[79]	hydrothermal	sodium citrate, Triethylenetetramine, Rose bengal	180 °C, 5 h	2.8	440/525
[80]	solvothermal	formamide, citric acid	-	3.51	457/643
[81]	solvothermal	p-PD, folic acid	200 °C, 2 h	5.65	rufous/red
[82]	solvothermal	biomass-cabbage	85 °C, 24 h; 75 °C, 4 h	3.4	500/678
[83]	solvothermal	biomass-kiwi fruit	100 °C, 20 h; 75 °C, 4 h	8.5	471/671
[84]	solvothermal	biomass-red tea	180 °C, 1 h	2.9	478/671
[63]	solvothermal	*o*-PD, sorbitol	200 °C, 12 h	4.36	597/645
[66]	solvothermal	*o*-PD, L-arginine, H_2_SO_4_	200 °C, 12 h	3.26	468/628
[67]	solvothermal	L-arginine, DL-malic acid	195 °C, 2 h	5.6	445/514
[57]	solvothermal	*o*-PD, ethanolamine	220 °C, 10 h	2.5	430/550
[65]	solvothermal	*o*-PD, L-cystine, ethanol	220 °C, 12 h	2.97	595/648
[56]	solvothermal	formamide, glutathione	160 °C, 1 h	2.8	460/683
[38]	microwave	*o*-PD	700 W, 20 min	7.65	360/530
[58]	microwave	phloroglucinol dihydrate, ethylenediamine, boric acid	400 W, 15 min	3.80	484/585
[58]	microwave	phloroglucinol dihydrate, ethylenediamine, boric acid	400 W, 15 min	2.85	484/565
[59]	solvent-free	ammonium citrate	180 °C, 1 h	6.8	462/560
[60]	solvent-free	*o*-PD, Al(NO_3_)_3_·9H_2_O	200 °C, 12 h	10	600/650

Acid can directly affect the pH value, and it is well known that pH value plays a critical role in the polymerization reaction. Similarly, during the carbonization process, pH significantly impacts the CDs’ properties, owing to the catalysis effect and the various existing forms of the functional groups attached to the surface of CDs. In addition, acids can affect the optical properties by doping heteroatoms, including O, S, and P [71,72]. For example, Song et al. obtained DE-CDs by heating *o*-PD and phosphoric acid at 200 °C for 24 h [72]. Two emission peaks at 440 and 624 nm were observed when excited at 380 nm. However, only one emission peak was observed when using *o*-PD as the sole raw material, which demonstrates the critical role of phosphoric acid. To further verify this, two more control CDs were prepared by mixing *o*-PD with trisodium phosphate or ammonium phosphate as raw materials. Both control particles showed two emission peaks at short wavelengths, demonstrating that the phosphorus precursor played a key role in controlling the dual-emission property of CDs. Moreover, acids can also be used as part of carbon sources, including citric acid, oxalic acid, gallic acid, and folic acid [73,74,80,81].

Biomass is a complex, heterogeneous, renewable, and environmentally friendly organic material. However, some current biomass is treated as waste, discarded, and landfilled, causing numerous environmental problems and directly threatening human health. In recent years, many researchers have started to use biomass as raw materials and have established green and low-cost solvothermal methods to synthesize DE-CDs. For example, Long et al. prepared DE-CDs from cabbage via the one-pot solvothermal method [82]. Under a single excitation of 410 nm, as-prepared DE-CDs exhibit two different emissions at 500 and 678 nm. Tong et al. prepared DE-CDs through the solvothermal method using kiwi fruit as a raw material [83]. As shown in Figure 3d, the synthesized DE-CDs exhibit two emissions at 471 nm (blue channel) and 671 nm (red channel), respectively, which can simultaneously monitor the activities of serum γ-glutamyl transpeptidase and alkaline phosphatase. Song and co-workers also synthesized DE-CDs via the simple one-step solvothermal treatment of black tea, and the synthesized DE-CDs had a robust red fluorescence emission peak at 671 nm and a weak blue fluorescence emission peak at 478 nm [84].

This section provided an overview of the different fabrication methods and precursors used in DE-CDs synthesis. As far as we know, the published DE-CDs can be obtained from the “bottom-up” approaches, including hydrothermal, solvothermal, microwave-assisted, and solvent-free carbonization processes. Table 2 summarizes and tabulates the advantages and disadvantages of these methods. For precursors, various molecules can be used, which can further be classified as aromatic small molecules, amino acids, acids, and biomass. Most of the precursors play multiple roles during the synthesis process. For example, *o*-PD, one of the most commonly used precursors, was used as a carbon and nitrogen source, which can also help form abundant amino groups exposed on the surface of DE-CDs.

## 3. Photoluminescence Mechanism of DE-CDs

Because various CDs can be synthesized through different routes using multiple raw materials, the PL mechanism of CDs is still under debate among researchers. Understanding the basic PL mechanism of CDs would help guide the development of effective synthesis routes and novel applications.

### 3.1. PL Mechanism of CDs

Currently, two types of mechanisms for CDs’ PL mechanism were mainly discussed. The quantum confinement effect, also called the size effect, is a widely accepted mechanism. Li et al. synthesized CDs with different particle sizes via the one-step alkali metal-assisted electrochemical method [85]. They interestingly found that the PL properties were closely related to the particle size: the small-size CDs (1.2 nm, center) emit UV light, the medium-size CDs (1.5–3 nm) give visible light emission, and the large-size CDs (3.8 nm) give near-infrared emission. However, many situations violate this dependence. Rhee and Kwon developed a size-controlled method using a “water-in-oil” emulsion as a self-assembled soft template [86,87]. They found that the PL peak position of different-size CDs was blue-shifted upon increasing the size of the CDs. This may be explained as the “size” not being the actual physical particle size but rather reflecting the sp^2^ (graphene) domain, and some CDs may embed many isolated sp^2^ clusters into the sp^3^ carbon matrix. Additionally, some other PL-affecting effects may also influence or dominate the PL properties.

The surface state also plays a critical role in the PL process. Changes in the surface structure, including polymers, defects, functional groups, and edge states, can directly affect the PL properties by altering the CDs’ electron energy level or light-emitting sites. It is well-known that pH can directly affect CDs’ PL properties, which is caused by the protonation and deprotonation process on the surface of CDs. A few years ago, our group obtained CDs, and we found that the PL spectrum of CDs significantly shifted from 445 to 565 nm when Cr^6+^ was added into the solution, which may have caused the formation of the improved rigidity and enlarged π conjugated defect structure after Cr^6+^ coordination [3]. In addition, a higher degree of surface oxidation or other effective modification can result in more surface defects and a red-shifted emission [43]. Consequently, the tunable PL properties of CDs can be achieved by either controlling the size of the sp^2^ domain or altering the chemical groups formed on the surfaces.

### 3.2. PL Mechanism of DE-CDs

The above-discussed PL mechanisms are independent; synergistic effects sometimes generate the dual-emission phenomenon. Wan and co-workers synthesized S and N co-doped DE-CDs through a simple hydrothermal method [71]. Dual emissions at 360 and 520 nm were observed when excited at the single wavelength, and the author claims that the yellow emission at 520 nm is from the intrinsic state emission, while the blue emission centered at 360 nm is generated from the surface energy traps. This mechanism was confirmed by the different optical properties of two control materials: silica-coated CDs and light conversion film (LCF). The silica-coated CDs were synthesized by a covalently linked silica overlayer on the surface of CDs. Due to the surface state effect loss, as-synthesized silica-coated CDs exhibit only one emission band centered at 520 nm. Additionally, the author also prepared the LCF by simply mixing S, N-C-dots, and PVA. The results show that the LCF offers similar dual emission centered at 360 and 520 nm; however, the intensity at 360 nm decreased significantly compared with S, N-C-dots. One possible reason could be that some functional groups of S, N-C-dots and the hydroxyl groups of PVA formed hydrogen bonds, significantly reducing the influence of surface states. These results demonstrate that the 360 and 520 nm dual emissions were generated from the surface energy traps and intrinsic state emission, respectively.

**Figure 4:**
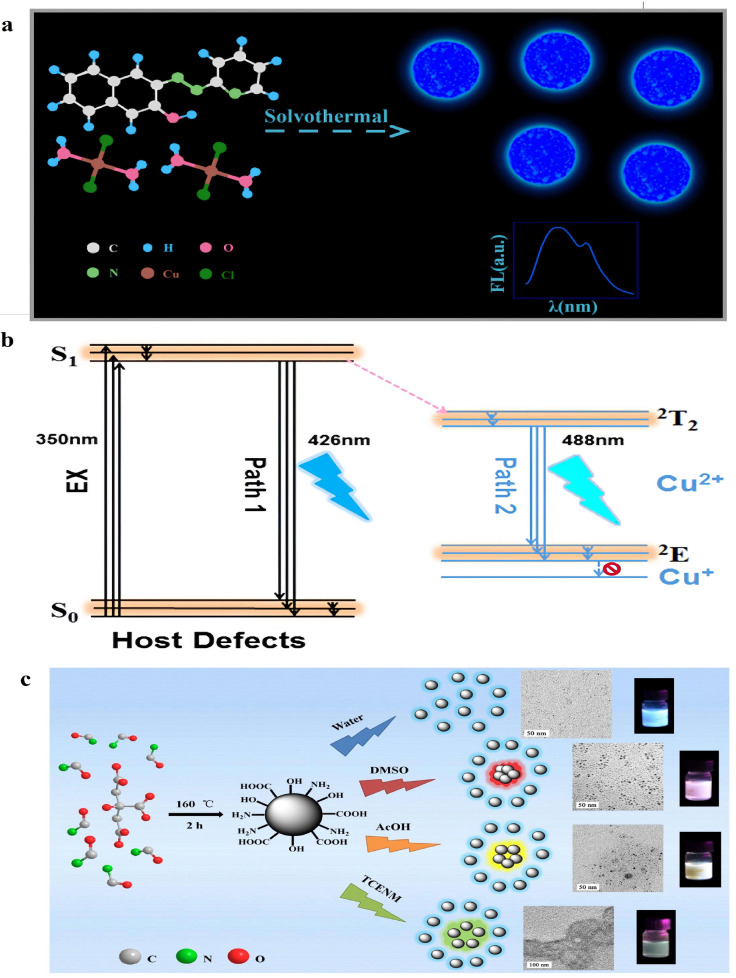
(**a**) Synthesized Cu-doped DE-CDs through a rapid and simple one-pot solvothermal method. Reproduced with permission from ref. [88], copyright 2018, The American Chemical Society. (**b**) The PL mechanism of the Cu-doped DE-CDs. Reproduced with permission from ref. [88], copyright 2018, The American Chemical Society. (**c**) The external effects of distance-dependent PL properties caused the dual emission. Reproduced with permission from ref. [80], copyright 2019, The Elsevier Inc.

In addition, the co-doped atom may also trigger the generation of dual emission. Zhu et al. synthesized Cu-doped DE-CDs via a rapid and simple one-pot solvothermal method [88]. As shown in Figure 4a, the dual-emission property of CDs mainly originates from π*-n transition (surface states) of the surface-attached functionalities. The emission at 426 nm was caused by the energy gap of CD host defects, while the doped Cu^2+^ provides a configuration related to another d-d orbital energy gap, in which valence electrons can transit easily (Figure 4b). The chelation of unpaired electrons could promote the transfer of the excited electrons of surface defects to the excited state of Cu^2+^ and then transit them to the ground state; therefore, a new electronic transition occurs, which results in new fluorescence emission at 488 nm.

Moreover, the external effects may also help the formation of DE-CDs. Wu and co-workers synthesized DE-CDs, which display dual emission located at 457 and 643 nm [80]. The authors claim that the signal at 457 nm belongs to the original emission of the CDs. In comparison, the red emission at 643 nm is independent of the excitation wavelength and can be manipulated by changing the polarity of the solvent (solvatochromic phenomenon, Figure 4c). After being proofed by Transmission Electron Microscope images, the author interestingly found that with the decrease in the polarity of the solvent, the distance between the CD particles was shortened. The second emission band at 643 nm was also red-shifted, caused by the Förster resonance energy transfer and re-absorption. A similar phenomenon was also found by Yoo [89]. As we know, the increased concentration of CD particles would directly lead to decreased interparticle distance between CDs. These changes may directly affect the PL properties of CDs. The long CD interparticle space leads to blue-emissive states, while the proximate distance of the CD is favorable for red emission, showing good agreement with the results from Wu [80].

Although the detailed PL mechanism behind DE-CDs is still an open topic for discussion, current studies provide crucial clues for further investigation. In this section, we have summarized the proposed PL mechanism of DE-CDs, and mainly three mechanisms have been discussed: (a) the dual emission could be separately generated by the intrinsic state emission and surface energy traps; (b) the co-doped atom may trigger the generation of dual emission by providing another orbital energy gap, in which valence electrons can transit easily; (c) besides the original emission of CDs, the interparticle distance between CDs can also affect their optical properties, in which long CD interparticle distance leads to blue-emissive states, while the proximate distance of the CDs is favorable for red emission.

## 4. Application of DE-CDs

### 4.1. Metal Cation Detection

**Figure 5:**
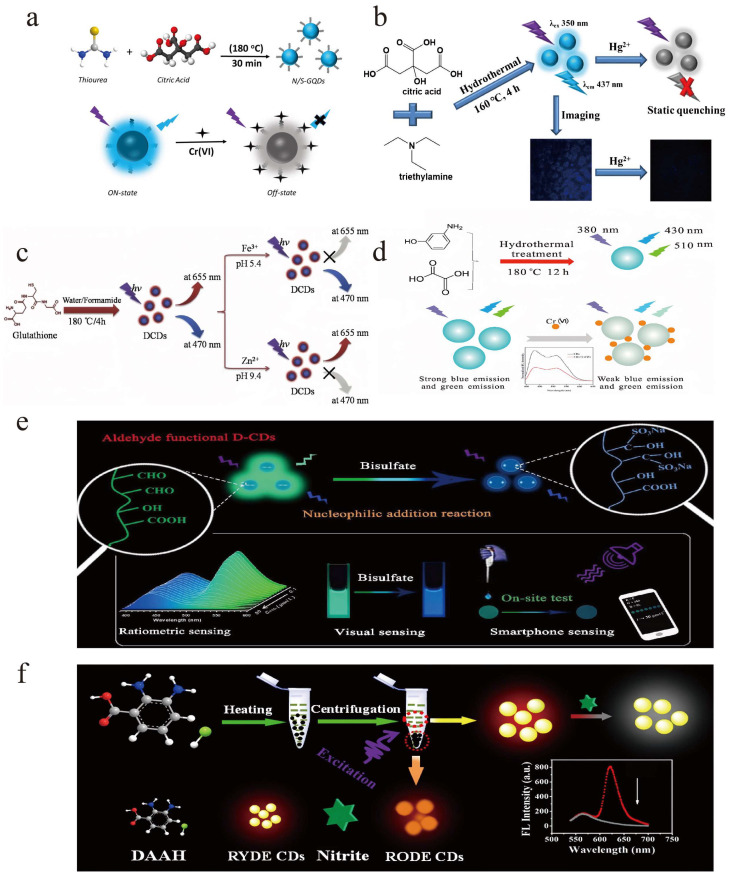
(**a**) One-step solid-phase pyrolysis synthesis of N/S-GQDs and their application on the on-off sensing procedure for Cr^6+^. Reproduced with permission from ref. [90], copyright 2023, The American Chemical Society. (**b**) Hydrothermal synthesis of CDs and their application in the on-off Hg^2+^ sensor. Reproduced with permission from ref. [91], copyright 2019, The Elsevier Inc. (**c**) Schematic illustration of the preparation of DE-CDs and the application in the detection of Fe^3+^ and Zn^2+^. Reproduced with permission from ref. [37], copyright 2018, The Elsevier B.V. (**d**) Hydrothermal synthesis DE-CDs and their ratiometric detection of Cr^6+^ based on the IFE mechanism. Reproduced with permission from ref. [92], copyright 2018, The Elsevier B.V. (**e**) Fabrication of aldehyde-functionalized DE-CDs and the principle and application of ratiometric detection of HSO_3_^−^. Reproduced with permission from ref. [93], copyright 2022, The Elsevier Ltd. (**f**) Preparation of red DE-CDs by a one-step pyrolysis method and nitrite could significantly reduce and quench the fluorescence peak at 621 nm. Reproduced with permission from ref. [94], copyright 2019, The American Chemical Society.

With the rapid development of the industry, industrial sewage usually carries different kinds of metal when discharged, including chromium, iron, zinc, copper, etc. These metal cations participate in the whole water circulation process and contaminate the environmental water. Some heavy metal elements may accumulate inside the human body, seriously endangering human health. Therefore, there is a high demand to establish reliable, sensitive, and selective methods for metal cation analysis. It is well known that CDs can detect many metal cations through the “on-off” strategy. In a typical “on-off” strategy, as shown in Figure 5a,b, the interaction between metal cations and CDs would inevitably lead to the quenching of CDs’ fluorescence intensity, and the decrease in the signal is inversely proportional to the concentration of the metal cations [90,91]. Therefore, the concentration of metal cations can be determined by the linear relationship with the quenched signal. This quenching phenomenon may be attributed to the photoinduced electron transfer (PET) mechanism. CDs serve as electron donors and metal cations as electron acceptors; therefore, electron transfer occurs and fewer excited electrons are returning from excited states to the ground state, causing the quenching of CDs. This phenomenon also occurs in the reaction between DE-CDs and metal cations. Because the results were calculated from the signal from at least two wavelengths, a more sensitive and accurate sensor can be facilely designed, which may effectively eliminate the background interferences and fluctuations.

Zhang et al. synthesized N/S co-doped DE-CDs via the hydrothermal method, and rare red/orange dual emission peaks (595 and 648 nm) were observed under single excitation [65]. Owning to the doped N and S elements, as-synthesized DE-CDs exhibited a high quantum yield of 35.7%. In the presence of Ag^+^, DE-CDs showed obvious fluorescence quenching at 595 and 648 nm quickly. This obvious fluorescence intensity change can be attributed to the fact that Ag^+^ can facilitate electron/hole recombination annihilation through the PET process, leading to changes in the surface electronic state of CDs. This proposed assay exhibits a good detection ability for Ag^+^ in the concentration range of 0~100 μM, showing the potential to expand their sensing application in environmental and biomedical fields. By using a similar quenching mechanism, Wang et al. prepared DE-CDs and established a ratiometric sensor for the detection of iron ions (Fe^3+^) and zinc ions (Zn^2+^) in different pH environments [37]. The DE-CDs were first synthesized by carbonizing glutathione in a water/formamide mixture, as shown in Figure 5c; the synthesized DE-CDs show dual-emission bands at 470 and 655 nm. Interestingly, Fe^3+^ ions could significantly quench the fluorescence intensity at 655 nm in an acidic environment, while Zn^2+^ ions can quench the signal at 470 nm under alkaline conditions. Both quenching phenomena were due to the effective PET process. The linear range was detected to be 2.5–30 μM/2.5–50 μM and the detection limit for Fe^3+^ and Zn^2+^ ions was calculated to be 0.8 μM and 1.2 μM, respectively. Therefore, as-synthesized DE-CDs could be used to detect Fe^3+^ ions and Zn^2+^ ions at different pHs.

Metal cations could also quench the fluorescence signal through other mechanisms, including the inner filtering effect (IFE). Unlike PET quenching, the fluorescence signal is not “quenched” in an IFE process instead of “blocked” during the detection process. Typically, CDs’ emission/excitation spectra overlap with the absorption spectra of the analytes (i.e., metal cations). Therefore, the analytes absorb the fluorescence signal before reaching the detector, causing a decrease in fluorescence intensity. Ma and co-workers synthesized dual-emitting carbon dots via the hydrothermal method using m-aminophenol and oxalic acid as raw materials [92]. Under the single excitation wavelength of 380 nm, the prepared DE-CDs exhibit dual-emission fluorescence peaks at 430 and 510 nm, and Cr^6+^ can significantly quench both signals (Figure 5d). Because Cr^6+^ exhibited broad absorption peaks centered at 260, 360, and 450 nm and the broad absorption spectrum of Cr^6+^ almost fully covered that of the DE-CDs, effective IFE occurs. The fluorescence signal was significantly quenched with the increase in Cr^6+^ concentration. Under the optimum conditions, the detection limit was calculated to be 0.4 μM, and the linear range was detected to be 2~300 μM. Moreover, the prepared fluorescent probes have been successfully applied for the analysis of Cr^6+^ in textile, steel, and industrial wastewater samples.

Except for quenching, metal cations sometimes could also enhance the fluorescence intensity of DE-CDs. Huang et al. prepared fluorescent DE-CDs through the microwave-assisted treatment of *o*-PD [38]. As-synthesized DE-CDs show an intrinsic dual emission at 360 and 530 nm under excitation of 320 nm. The DE-CDs could effectively capture Cu^2+^ via coordinating with the nitrogen lone pair of -NH_2_ to form a complex (Cu^2+^-*o*PD) by electron pair sharing. The as-formed complex can effectively quench the fluorescence signal at 360 nm, while the signal at 530 nm is gradually intensified. The enhanced fluorescence intensity at 530 nm could account for the inhibition of PET by forming the complex of Cu^2+^ with -NH_2_ on the CDs surface. Therefore, by monitoring the ratio of the fluorescence intensity F_360_/F_530_, a ratiometric fluorescent sensor for Cu^2+^ detection was established.

### 4.2. Food Safety Analysis

DE-CDs have also been applied in food safety analysis. Nowadays, people’s living standards and health awareness have been continuously improved. Food is the most basic requirement in daily life, and the public are paying more and more attention to the food safety problem. Food safety problems can arise from different aspects, including environmental pathogens, food processing hazards, food toxins, and harmful pesticide residues in agricultural products. To solve this problem, practical food safety analysis is the key to ensuring food safety. Therefore, applying novel detection techniques in food safety testing is urgent.

It is well known that HSO_3_^−^ is widely used as an antioxidant and preservative, while excessive use can harm human health. To analyze HSO_3_^−^, Deng et al. synthesized aldehyde-functionalized DE-CDs via the hydrothermal method by using 2,4-dihydroxybenzaldehyde and NaOH as raw materials [93]. Under excitation of 360 nm, the DE-CDs showed dual-emission characteristics at 435 and 520 nm. Through the nucleophilic addition reaction between HSO_3_^−^ and the aldehyde group on the surface of DE-CDs, the fluorescence intensity at 520 nm decreases, whereas that at 435 nm remains stable; meanwhile, the fluorescence color was significantly changed from green to dark blue. Therefore, a ratiometric sensor for HSO_3_^−^ was established, and the detection limit was calculated to be 42 nM, which could be successfully applied for real-time monitoring of HSO_3_^−^ in food. Moreover, a smartphone sensing platform is designed to simplify the detection process, which provides a convenient visual sensing tool for real-time monitoring of HSO_3_^−^ in food. Figure 5e schematic illustrates the preparation of DE-CDs and the detection principle of HSO_3_^−^. Similarly, Liu et al. prepared unmodified and long-wavelength red/yellow DE-CDs for trace nitrite analysis [94]. The DE-CDs were synthesized through the one-pot hydrothermal route by using 2,3-diaminobenzoic acid hydrochloride as raw materials. As shown in Figure 5f, adding nitrite could significantly reduce and quench the fluorescence peak at 621 nm, while the signal at 566 nm remains unchanged. Under the optimal conditions, the detection limit of nitrite was calculated to be 31.61 nM, and this nanoprobe can be successfully applied to the analysis of nitrite in bacon, sausage, pickle, and milk samples.

Wang et al. prepared a dual-channel tea polyphenols (TPPs) sensor based on deep ultraviolet DE-CDs, which were synthesized by using guanidine as the sole starting material [95]. The enrichment-specific hydroxyl sites (such as-NH_2_ and -COOH) of DE-CDs can specifically react with the phenolic hydroxyl groups of TPPs to generate dynamic amide and carboxylic acid bonds through dehydration and/or condensation reactions. As a consequence, the fluorescence properties of DE-CDs are changed and the ratio of fluorescence signal at 297 and 395 nm (F_297nm_/F_395nm_) of DE-CDs is decreased with the increase in TPPs concentration, which can be used for the construction of fluorescence ratiometric sensors for TPPs analysis. In addition, the adsorption at 320 nm is significantly increased when DE-CDs are exposed to the TPPs environment. Therefore, as-prepared DE-CDs can be successfully applied to the dual-channel determination of TPPs in tea samples, showing great potential application prospects in food analysis.

### 4.3. Biosensing and Cell Imaging

#### 4.3.1. Biosensing

The past decade has seen a proliferation in life science. Investigating the fundamental processes in the life sciences heavily relies on the fast, sensitive, and reliable detection of the interplay of different biomolecules. Owning to the merits of CDs, including a small size (1–10 nm), good water solubility, excellent biocompatibility, unique optical properties, easy functionalization, and low toxicity, DE-CDs are widely used in biosensing and cell imaging (Table 3). Generally, the sensing principle is based on the specific reaction between the target and the functional groups on the surface of DE-CDs. This specific interaction may lead to any change in DE-CDs’ optical properties, including emission intensity, emission wavelength, or lifetime, which can be employed as measurable signals for analyte analysis.

**Figure 6:**
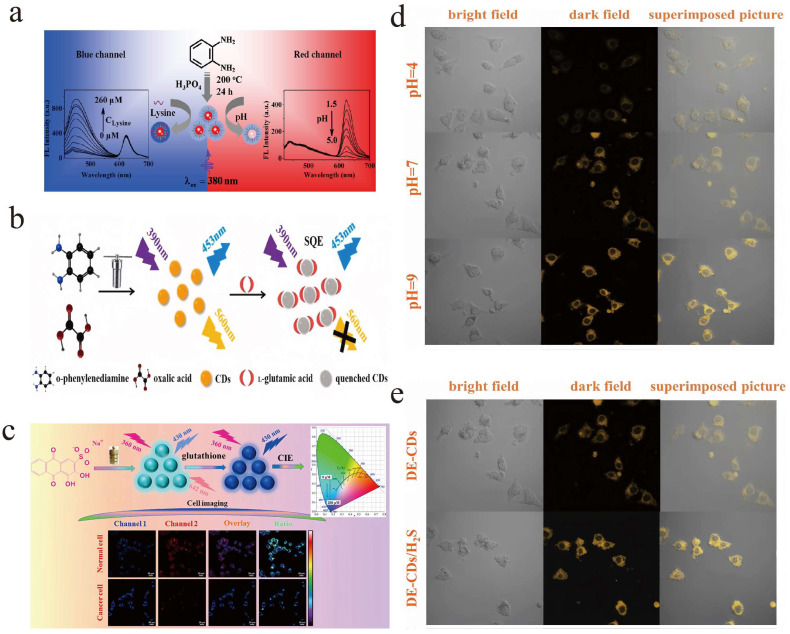
(**a**) Diagram of the preparation procedure of DE-CDs and specific ratiometric detection of lysine and pH. Reproduced with permission from ref. [72], copyright 2017, The American Chemical Society. (**b**) Hydrothermal synthesis of DE-CDs and their application as ratiometric sensors for L-Glu. Reproduced with permission from ref. [73], copyright 2022, The Elsevier. (**c**) DE-CDs were synthesized, and these particles were further applied in the ratiometric detection of glutathione and distinguishing cancer cells from normal cells. Reproduced with permission from ref. [99], copyright 2020, The American Chemical Society. (**d**,**e**) One-pot hydrothermal strategy was used to synthesize DE-CDs, which were further applied as pH sensors to image the pH in Hela cells and zebrafish, respectively. Reproduced with permission from ref. [104], copyright 2023, The Elsevier B.V.

Song et al. prepared functional DE-CDs using a the single-pot hydrothermal carbonization method. When excited at 380 nm, DE-CDs exhibit two different fluorescence emission peaks at 440 and 624 nm [72]. Lysine can significantly enhance the fluorescence intensity at 440 nm. In comparison, the peak at 624 nm remains stable. The color of the solution gradually changes from pink to purple after adding lysine (Figure 6a). Based on this, a ratiometric sensor for lysine was established, which could be used to quantitatively monitor the changes in lysine concentration in living cells, showing great potential for practical application value in the diagnosis of lysine-related diseases and disorders. Similarly, Yuan et al. synthesized DE-CDs through the one-pot hydrothermal method using *o*-PD and oxalic acid as raw materials [73]. As shown in Figure 6b, the synthesized DE-CDs showed two fluorescence emission peaks at 453 and 560 nm when excited at 390 nm. The presence of L-Glu weakens the fluorescence at 560 nm, while the signal at 453 nm remains unchanged. Therefore, the signal at 453 and 560 nm can be used as the reference signal and responsive signal. By using this method, the L-Glu in fetal bovine serum was successfully analyzed with satisfied recovery.

#### 4.3.2. pH Sensor

The pH value is one of the most important parameters of the cells and plays a critical role in many cellular events, including proliferation, migration, apoptosis, and so on. An abnormal pH value directly leads to inappropriate cell function, growth, and division. Therefore, to understand the status of the cells, it is crucial to detect the pH value. Compared with other pH sensors, the optical properties of DE-CDs are determined by the structure of functional groups on the surface, which are directly connected to the pH value through the protonation and deprotonation process. Therefore, DE-CDs are an ideal cellular pH sensor.

Xia et al. reported intrinsic DE-CDs for proportional pH sensing [98]. The two emission bands at 393 and 580 nm showed strong pH dependence. Under the excitation at 365 nm and the pH value change from 8.0 to 2.2, the intensity at 393 nm gradually increased, while the signal at 580 nm decreased significantly. This sensor exhibits the merits of a fast response time, high sensitivity, good reversibility, and high accuracy, indicating that the prepared DE-CDs can be used as an excellent ratiometric luminescent nanoprobe for pH detection. Ghadareh et al. also prepared DE-CDs, and as-prepared CDs were used as ultra-bright fluorescent probes to monitor the pH value ranging from 2.5 to 12.0 with high sensitivity [97]. This work provides an effective optical probe for intracellular pH sensing and multicolor imaging of living HeLa and MDA-MB-231 cells.

#### 4.3.3. Cell Imaging

Bioimaging enables an understanding of the structure and physiological functions of cells and organisms. Therefore, it is a heavily relied upon tool in the healthcare sector for diagnosing human diseases. CD-based bioimaging methods are attracting increasing attention since CDs show good water solubility, excellent biocompatibility, unique optical properties, etc. DE-CDs perform better quantification abilities than single-emitting CDs; however, most reported DE-CDs emit blue or green fluorescence at an effective excitation wavelength. Due to the ubiquitous bio-related autofluorescence interference, the preparation of red DE-CDs for proportional detection and bioimaging remains challenging.

Li et al. reported a simple strategy for manufacturing dual-emission carbon nanodots and demonstrated its application in the ratiometric detection of glutathione and distinguishing cancer cells from normal cells [99]. Using alizarin carmine as the carbon source, DE-CDs were synthesized by simple hydrothermal treatment, showing interesting dual-emission behavior at 430 and 642 nm (Figure 6c). With the increase in GSH concentration, the fluorescence signal at 430 nm increased, whereas that at 642 nm decreased slightly. An effective platform for ratiometric GSH sensing was established by monitoring the intrinsic ratio (I430 nm/I642 nm). The cytotoxicity and biocompatibility of as-synthesized DE-CDs were evaluated. Moreover, it can be used for cell imaging research and to discriminate SMMC-7721 (cancer cells) from L02 (normal cells) since the GSH content of cancer cells is significantly higher than that of corresponding normal cells.

Hu et al. prepared carbon dots with good water solubility, biocompatibility, and excitation-independent dual emission (two PL emission peaks at 630 and 680 nm, respectively) via the hydrothermal treatment of dicyandiamide and *o*-PD in dilute sulfuric acid [100]. The synthesized DE-CDs were stained with Hela cells to evaluate their potential imaging ability. The results show that most HeLa cells survived, and strong red fluorescence could be observed, indicating the potential application of CDs in bioimaging. Meng and co-workers synthesized the DE-CDs through a one-pot hydrothermal strategy using neutral red and sodium 1,4-dinitrobenzene sulfonate as raw materials [104]. As synthesized DE-CDs can be used as an effective ratiometric sensor for H_2_S; additonally, the fluorescence signal also increased as the pH value increased from 2.0 to 10.2. As shown in Figure 6d,e, the synthesized DE-CDs can be used as imaging agents for pH sensing in living cells and zebrafish.

### 4.4. Optoelectronic Devices

Light-emitting diodes (LEDs) are widely used in our daily lives and have been regarded as evolutionary innovations in lighting [16]. Based on the low toxicity, high stability, and excellent optical properties of CDs, DE-CDs have been applied in LED preparation. Guan et al. reported a simple one-step solvothermal synthesis of nitrogen and sulfur co-doped DE-CDs [103]. Because of the high fluorescence efficiency, as-prepared DE-CDs were applied for the preparation of bright and stable B-LEDs, G-LEDs, R-LEDs, and WLEDs, which were prepared from mixtures with various ratios of different CDs and DE-CDs. The results show that as-prepared LEDs exhibit excellent PL properties and enhanced stability.

The numerous merits of DE-CDs trigger their wide application in many fields. DE-CDs contain different functional groups, providing an ideal analyte-binding site. When the analyte conjugates on the surface of the DE-CDs, the surface structure is inevitably changed, leading to a change in PL properties. Therefore, the dual emission can be used as a responsive and control signal, and a ratiometric sensor can be established. Currently, DE-CDs have already been successfully applied in metal cation detection, food safety analysis, biosensing, pH sensors, and cell imaging. In addition, thanks to their excellent optical properties, the application of DE-CDs in optoelectronic devices has also been reported.

## 5. Summary and Outlook

DE-CD-based ratiometric probes are advantageous for analytical detection since they overcome the disadvantages of single-wavelength-based sensors by minimizing the effects of the instrument, the environment, and the concentration of the receptor. This review has comprehensively provided an overview of commonly used methods and raw materials for the fabrication of DE-CDs. Then, the possible PL mechanism for CDs and DE-CDs has been discussed. Moreover, DE-CDs’ applications in metal cation detection, food safety analysis, biosensing, pH sensors, cell imaging, and optoelectronic devices have been well summarized.

Although ratiometric sensing can be achieved by physical mixing and the nanohybridization of CDs in combination with other PL materials, this inevitably requires complex preparation, separation, and purification. The one-step synthesis of DE-CDs and direct establishment of ratiometric fluorescent sensors allows the rapid, sensitive, and accurate detection of target molecules. Currently, a number of DE-CDs have been synthesized and successfully applied in different fields, but some challenges still need to be addressed. (1) There is still a lack of high-quality methods to synthesize DE-CDs. Although many DE-CDs have been synthesized through hydrothermal/solvothermal and microwave-assisted methods using different raw materials, it is still challenging to synthesize CDs with dual emissions. Therefore, there is still a high demand for high-quality methods to synthesize DE-CDs. (2) The mechanism behind the dual emission of CDs is still unclear, and the ambiguous mechanism of CDs directly causes difficulty in predicting or controlling the synthesis of CDs with a specific emission. (3) Most of the existing DE-CDs exhibit their emission centered in the shorter wavelength region, significantly restricting their application in biomedical assays and therapy. Therefore, there is still a high demand to synthesize DE-CDs with near-infrared emissions. (4) The reproducibility of DE-CDs is somehow complicated, which calls for understanding the reaction mechanism of DE-CDs formation, including the nucleation mechanism and effects of different reaction conditions. Although many challenges occurred on the road to development, we believe these problems will be solved and DE-CDs will have a bright future based on their unique properties.

## Figures and Tables

**Table 2:** The advantages and disadvantages of various synthesis approaches.

Synthesis Approaches	Advantages	Disadvantages
Hydrothermal/Solvothermal	Simple, easy, and low-cost synthesis	Long reaction times and high-temperature treatment
Microwave	Short reaction times and high yields	Lack of heating uniformity and unsuitable for scale-up industrial production
Solvent-free	Simple, solvent-free, large-scale preparation	High-temperature treatment

**Table 3:** The various applications of DE-CDs.

Ref.	Size (nm)	Peaks (nm)	Applications	Linear Range	Detection Limits	Signal Readout
[37]	3.61	470/655	Fe^3+^/Zn^2+^	2.5~30 µM/2.5~50 µM	0.8/1.2 μM	F_470_/F_655_
[38]	7.65	360/530	Cu^2+^	0.8~55 μM	44.63 nM	F_360_/F_530_
[56]	2.8	460/683	Zn^2+^/Mn^2+^/Cu^2+^	0.005~8/0.0001~10/0.0001~50 µM	9.64/3.24/1.7 nM	F_683_/F_652_
[72]	5	440/624	Lysine/pH	0.5~260 mΜ/1.5~5.0	94 nM/-	F_440_/F_624_
[73]	3.29	453/560	L-Glu	0~200 μM; 200~400 μM	0.085 μM	F_560_/F_453_
[65]	2.97	595/648	Ag^+^/pH/cell imaging	0~100 μM/1.0~13.0/-	0.4 μΜ/-/-	ΔF
[89]	2.22	431–500/650	wLED	-	-	-
[92]	3.2	430/510	Cr^6+^	2~300 μM	0.4 μM	F_510_/F_430_
[88]	3.72	426/488	Fe^3+^/vitamin A acetate/pH	0~4000 μM/-/6.09~11.70	-	(F_0-_F)/F_0_
[93]	2	435/520	HSO_3_^−^	0.1~30 μM	42 nM	F_435_/F_520_
[94]	4.6	566/621	nitrite	0.1~100 μM	31.61 nM	F_621_/F_566_
[95]	3	297/395	TPPs	5.0~100 µg/mL	3.5 ± 0.04 ng/mL	F_297_/F_395_
[96]	5.87	416/481	TM	0.10~20.00 μM	2.90 × 10^−6^ μM	F_416_/F_481_
[97]	2.88	393/580	pH	2.2~8.0; 2.2~4.0	-	F_393_/F_580_
[98]	3.5	336/540	pH/cell imaging	2.5~12.0/-	-	F_336_/F_540_
[99]	2.37	430/642	GSH/cell imaging	1~10 μM; 25 ~150 μM/-	0.26 μM/-	F_430_/F_642_
[100]	5.71	630/680	methyl blue/cell imaging	0.5~300 μM/-	0.43 μM/-	F_630_/F_680_
[101]	5.2	525/603	ONOO^−^/cell imaging	0.03~60 μM/-	11.6 nM/-	F_525_/F_603_
[102]	1.8	345/450	CTC/cell imaging	0.25~25.0 μM/-	16.45 nM/-	F_430_/F_345_
[103]	2.89	600/650	water in ethanol/wLEDs	0%~70%/-	-	ΔF

## Abbreviations

CDs	carbon dots
DE-CDs	dual-emission carbon dots
*o*-PD	*o*-phenylenediamine
PL	photoluminescence
QY	quantum yield
TNP	2, 4, 6-trinitrophenol
PET	photoinduced electron transfer
TPPs	tea polyphenols
QDs	quantum dots
GSH	glutathione
LCF	light conversion film
IFE	inner filtering effect
